# Simultaneous transvenous lead extraction and implantation of leadless cardiac pacemaker: A case report

**DOI:** 10.1002/ccr3.7223

**Published:** 2023-06-26

**Authors:** Mengwei Wu, Hao Su

**Affiliations:** ^1^ Department of Cardiology First Affiliated Hospital of University of Science and Technology of China (Anhui Provincial Hospital) Hefei China; ^2^ Wannan Medical College Wuhu China

**Keywords:** devices infection, lead extraction, leadless pacemaker, pacing dependence, venous infection

## Abstract

Cardiovascular implantable electronic device (CIED) infection is a serious complication and remains the most common indication for transvenous lead extraction (TLE). In addition, there are serious challenges such as venous access occlusion and reinfection after extraction. Leadless pacemaker (LP) provides a safe and effective pacing option for patients with device‐related infections. We describe here a case of simultaneous transvenous lead extraction and leadless pacemaker implantation due to bilateral venous infection and pacing dependency.

On February 1, 2020, a 64‐year‐old man who received a dual‐chamber pacemaker via the left subclavian vein on May 27, 2019, for sick sinus syndrome and third degree atrioventricular block developed an infection in his left pacemaker pocket without streaks of pus oozing from the pocket. He was subsequently hospitalized in our hospital on March 5, 2020, and the laboratory report showed elevated white blood cells and C‐reactive protein (CRP). Blood and pus cultures were negative, and there was no evidence of bloodstream infection. After 4 days of anti‐infection treatment, the original pacemaker was removed from the patient's body; 2 days later, a contralateral dual‐chamber pacemaker was implanted via the subclavian vein. The treatment was satisfactory, and the patient was discharged from the hospital.

On August 20, 2021, the patient again developed exposure of the right pacemaker and pocket erosion with streaks of pus exuding from the pocket, but no fever. Blood cultures indicated Staphylococcus epidermidis, pus cultures were negative, and laboratory results showed high a level of CRP (CRP >5 mg/dL). The patient was treated with vancomycin for 7 days. After physician discussion, the situation strongly indicated the necessity for the patient to undergo simultaneous technical operations of transvenous lead extraction (TLE) and implantation of a leadless pacemaker (LP). Blood cultures were negative within 72 h after extubation. At 9 months of observation, the patient had no subsequent recurrence of infection by echocardiography and blood tests.

## 1 PROCEDURAL DETAILS

**FIGURE 1:**
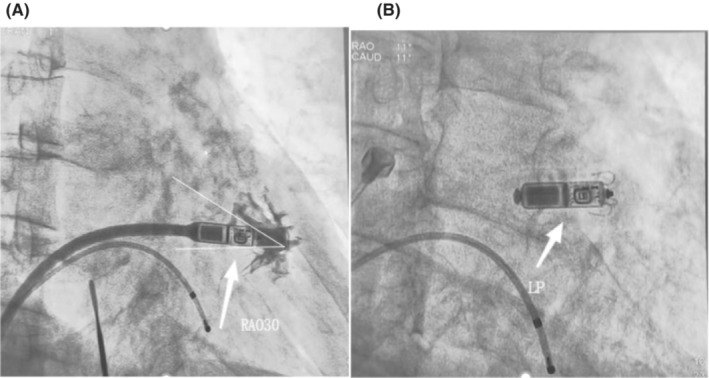
Tether, delivery system, and sheath were removed successively for the procedure completion. (A) Leadless pacing position, RAO30; (B) Micra leadless pacemaker successfully Released.

The procedure was performed in the cardiovascular catheter room of the First Affiliated Hospital of University of Science and Technology of China (Anhui Provincial Hospital). The patient was positioned in the supine position, and a temporary endocardial pacemaker was implanted through the left femoral vein. After local anesthesia, the pacemaker pulse generator was removed and then the right atrial and right ventricular electrodes were removed through the right subclavian vein by applying a locking wire (LR‐0FA01) and an 11F Evolution mechanical dilatation sheath (LR‐EVN‐9) (C00K,Corporation, USA). The right femoral vein was then punctured, successively dilated and implanted into a sheath (outer diameter 27F) via a leadless pacemaker delivery system (Micra TPS). The Micra device was positioned and released in the right ventricular septum by right anterior oblique and left anterior oblique positioning (avoiding the original right ventricular lead implantation site) after satisfactory results were obtained from electrical parameter testing. The measured pacing parameters included ventricular threshold, ventricular sensing, and impedance of 0.63 v, 10 mv, and 1040 ohms, respectively. The procedure was completed by sequentially removing the tether, delivery system, and sheath (Figure 1). The operation ended with the removal of the temporary pacemaker lead and sheath from the left femoral vein.

## 2 DISCUSSION

In recent years, the number of pacemaker implantations has been increasing with the development of pacing technology and the expansion of indications. The incidence of associated complications, especially pacemaker device infections, has been increasing.^1^ Since the removal or extraction of cardiovascular implantable electronic device (CIED) is usually mandated if infection occurs.^2^ However, there is a dispute about the optimal timing of device reimplantation following the infected CIED extraction.^3^ Current practice standards support waiting between 72 h and 14 days before reimplanting a device.^4^

For patients with pacemaker indications, a new pacemaker needs to be re‐implanted, and this process has many risks, such as reinfection and venous approach occlusion.^5^ These patients with potential infectious complications have a high risk of recurrent infection with conventional pacemakers implanted through the traditional venous approach. Patients with pacemaker dependency receive a temporary transvenous pacing lead while being treated for CIED infection. This strategy is associated with the risk of maintaining the original infection and increasing the likelihood of recurrent CIED infections.^6^ Leadless pacemakers (LP), as a new technology that integrates the pulse generator and the pacing lead, avoiding pocket and lead‐related complications, have become the implantation of choice after pacemaker device infection.^7^ There is emerging evidence that concurrent LP implantation and lead extraction is safe during active infection, especially in pacemaker‐dependent patients.^8^ Cases of infection after conventional pacemaker implantation are rare in the same patient. Few, if any, reports of extraction of the original device and simultaneous implantation of an LP have been published.

A previous study^9^ reported no recurrent infections in 17 patients who underwent LP implantation and CIED extraction during the same procedure, and 4 of them had positive blood cultures at the time of implantation. In addition, LP does not lead to reinfection even if the infected pacemaker system is removed prior to the same procedure.^6^ No evidence of CIED infection was reported after LP implantation or CIED system removal with a mean follow‐up of more than 2 years, in spite of an elevated risk of prior CIED infection and recurrent infection.^10^ This is consistent with the results we reported. Hence, LP may provide new opportunities for the management of patients with pacemaker infection and pacemaker dependence.

## 3 CONCLUSION

In this case, the patient was pacemaker dependent and at high risk for venous reinfection. Comprehensive considerations led us to choose to perform simultaneous electrode lead removal and leadless pacemaker implantation, with no significant postoperative complications after operation. Therefore, LP is preferred at the 9‐month follow‐up observation with no subsequent recurrence of infection by echocardiography and blood culture. Taken together, simultaneous leadless pacemaker implantation and CIED extraction are safe and feasible. This strategy may be particularly useful for patients dependent on pacemakers.

## AUTHOR CONTRIBUTIONS

**Mengwei Wu:** Writing – original draft. **Hao Su:** Project administration; supervision; writing – review and editing.

## FUNDING INFORMATION

This research did not receive any specific grant from funding agencies in the public, commercial, or not‐for‐profit sectors.

## CONFLICT OF INTEREST STATEMENT

The authors have no conflict of interest to disclose.

## INFORMED CONSENT

Written informed consent was obtained from the patient to publish this report in accordance with the journal's patient consent policy.

## Data Availability

The data underlying this article will be shared on reasonable request to the corresponding author.
